# Makroskopische und mikroskopische Veränderungen des N. vestibulocochlearis nach Gamma-Knife-Therapie

**DOI:** 10.1007/s00106-021-01104-2

**Published:** 2021-09-01

**Authors:** Maximilian Scheer, Christian Scheller, Julian Prell, Christian Mawrin, Torsten Rahne, Christian Strauss, Sebastian Simmermacher

**Affiliations:** 1 Klinik für Neurochirurgie, Universitätsklinikum Halle, Medizinische Fakultät der Martin-Luther-Universität Halle-Wittenberg, Ernst-Grube-Straße 40, 06120 Halle, Deutschland; 2 Institut für Neuropathologie, Universitätsklinikum Magdeburg, Magdeburg, Deutschland; 3 Klinik für Hals‑, Nasen‑, Ohrenheilkunde, Universitätsklinikum Halle, Halle, Deutschland

**Keywords:** Radiochirurgie, Vestibularisschwannom, Komplikation, Demyelinisierung, Fallbericht, Radiosurgery, Vestibular schwannoma, Complication, Demyelination, Case report

## Abstract

Wir berichten über einen Fall, bei dem makroskopische und mikroskopische Veränderungen des Verstibularnervs nach radiochirurgischer Behandlung eines intrameatalen Vestibularisschwannoms beobachtet wurden. Der Fallbericht zeigt das erste Mal ein morphologisches Korrelat der unerwünschten Effekte der Gamma-Knife-Therapie von Vestibularisschwannomen und unterstreicht, dass trotz eines deutlichen Abstands zum bestehenden Tumor degenerative Veränderungen der neuralen Strukturen erwartet werden können.

## Abstract (en)

We report on a case in which macroscopic and microscopic changes of the vestibulocochlear nerve could be observed after radiosurgery of an intrameatal vestibular schwannoma. This case shows for the first time a morphological correlate for undesirable effects after radiosurgical treatment of a vestibular schwannoma and indicates that despite a certain distance to the actual tumor, degenerative changes in neural structures can be expected.

## Falldarstellung

### Anamnese

**Figure 1:**
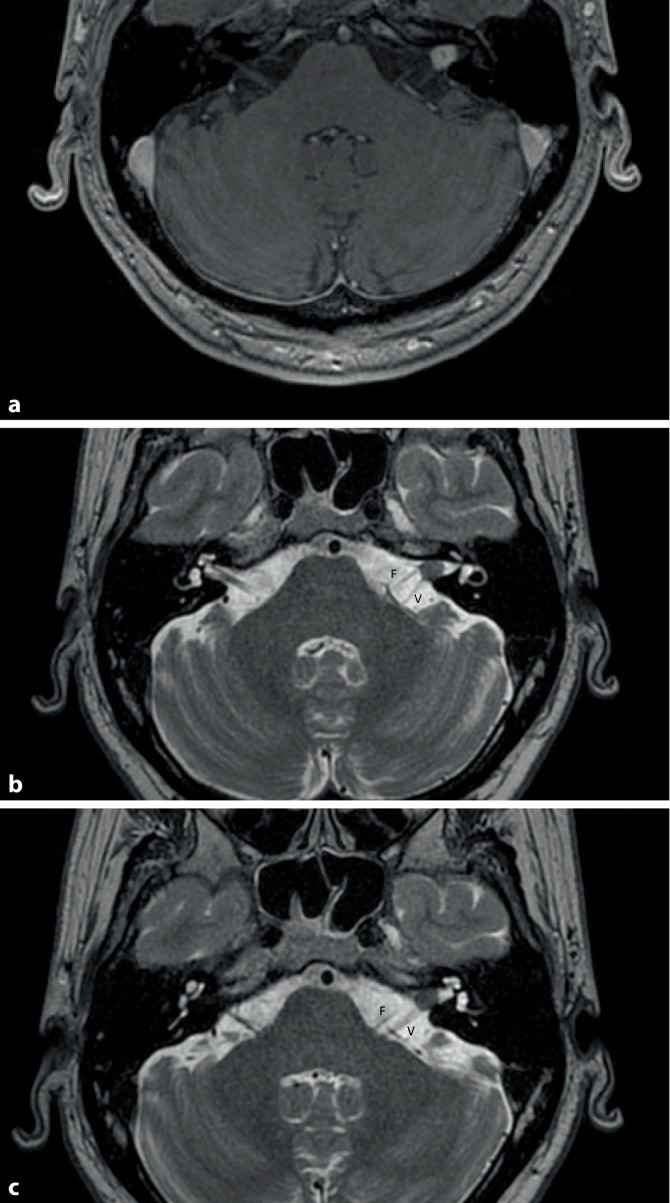

Bei einem 57-jährigen männlichen Patienten, welcher bis auf einen arteriellen Hypertonus gesund war, erfolgte drei Jahre vor der Konsultation in unserer Klinik eine Gamma-Knife-Therapie (13 Gy, Volumen 0,13 cm^3^, 65 % therapeutische Isodose, die Cochlea lag vollständig außerhalb der 6‑Gy-Isodose) eines intrameatalen Vestibularisschwannoms (Abb. 1). Vor der Gamma-Knife-Therapie beklagte der Patient einen progredienten Tinnitus sowie subjektiven Hörverlust über einen Zeitraum von knapp einem Jahr. Magnetresonanztomographie(MRT)-Verlaufskontrollen zeigten zunächst einen stabilen Befund. Nun berichtete der Patient von einem zunehmenden Tinnitus, einer Schwankschwindelsymptomatik, einer stetig zunehmenden Hörminderung, schmerzhaften Verkrampfungen der Gesichtsmuskulatur und einer Taubheit der linken Gesichtshälfte.

### Klinischer Befund

**Figure 2:**
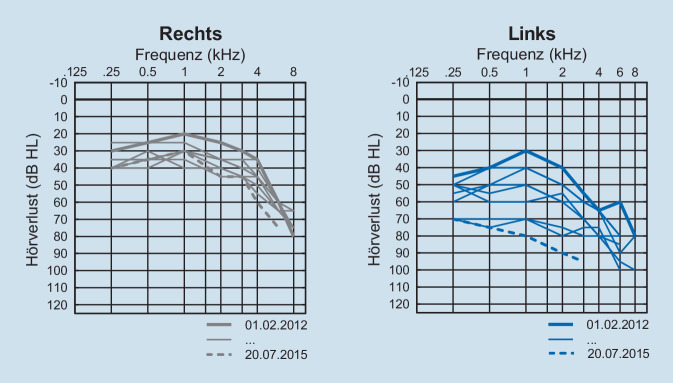

Es bestand ein Hemispasmus facialis, eine Hypästhesie im Versorgungsgebiet des N. trigeminus (V2 und V3) und ein pathologischer Unterberger-Tretversuch sowie ein positiver Romberg-Test. Audiologisch zeigte sich eine hochgradige Einschränkung des Hörvermögens. Dieses hatte sich im Laufe der letzten 3 Jahre allmählich verschlechtert (Abb. 2).

## Diagnose

### Kranielle Bildgebung

**Figure 3:**
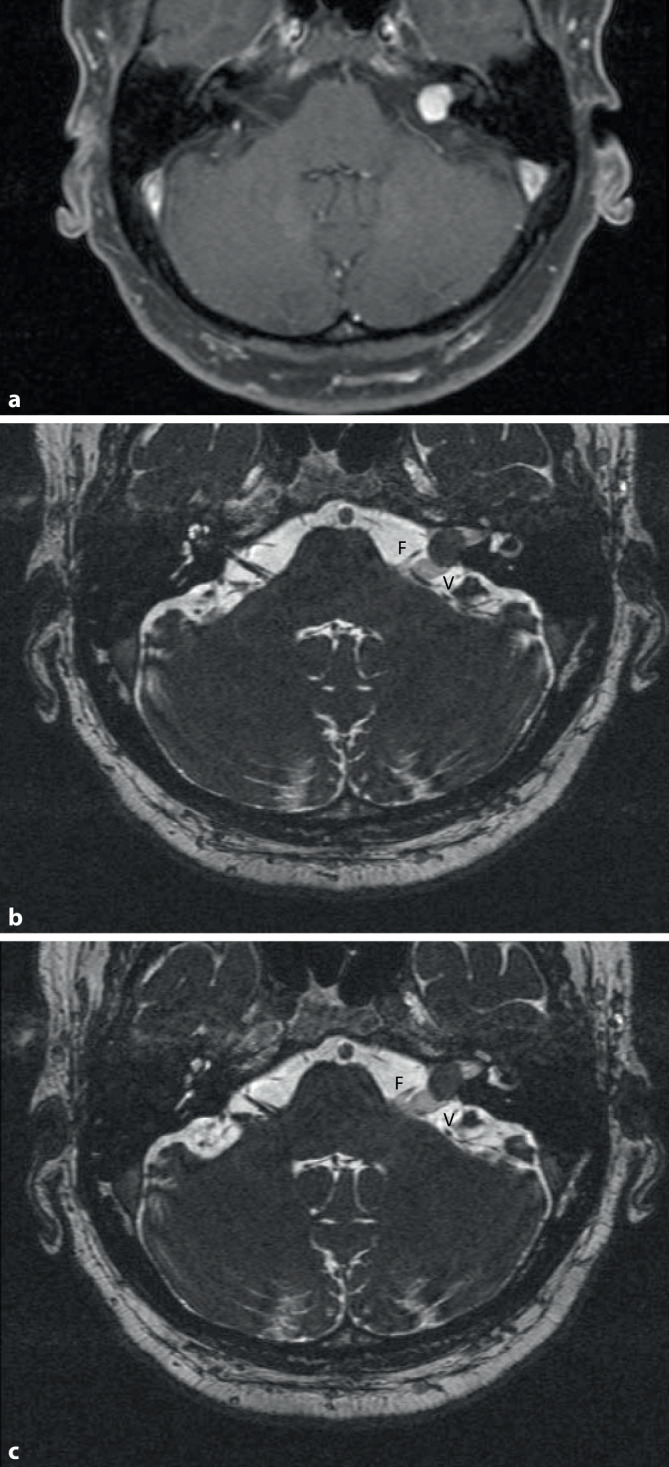

In der cMRT-Bildgebung zeigte sich im Vergleich zu den Voraufnahmen ein Progress des kontrastmittelaufnehmenden Tumoranteils im linken Kleinhirnbrückenwinkel. Ein Verlust der intrakapsulären Kontrastmittelaufnahme, wie es oft nach einer Radiotherapie beschrieben wird, kam nicht zur Darstellung (Abb. 3a) [10]. Zusätzlich zeigte sich eine massive Auftreibung des N. vestibulocochlearis und des N. facialis (Abb. 3b).

## Therapie und Verlauf

**Figure 4:**
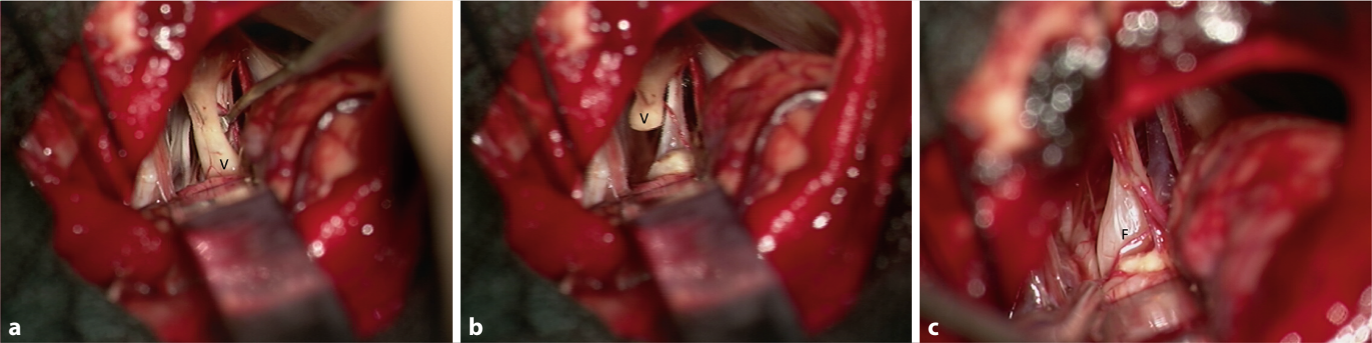

Aufgrund des klinischen und radiologischen Progresses wurde die Indikation zur mikrochirurgischen Tumorentfernung mit neurophysiologischem Monitoring gestellt. Perioperativ erfolgte die i.v.-Gabe des Kalziumantagonisten Nimodipin. Während der Operation über einen retrosigmoidalen Zugang imponierte der N. vestibulocochlearis proximal des eigentlichen Tumors als massiv aufgetrieben (Abb. 4a). Als Tumorursprung konnte der N. vestibularis inferior identifiziert werden. Intraoperativ kam es zu einer Verschlechterung der akustisch evozierten Potenziale (AEP). Bei bereits hochgradiger Einschränkung des Hörvermögens präoperativ entschloss man sich, den N. vestibulocochlearis zugunsten einer kompletten Tumorentfernung zu resezieren (Abb. 4b). Intraoperativ wurde ebenfalls eine kontinuierliche Elektromyographie (EMG) der vom N. facialis innervierten Muskulatur abgeleitet. Hier kam gehäuft zu A‑Train-Aktivität, auch ohne Manipulation des Nervs. Als Korrelat zu dieser Spontanaktivität im EMG zeigte sich der N. facialis intraoperativ an der Root-Exit-Zone ebenfalls massiv aufgetrieben (Abb. 4c). Ein Gefäß-Nerven-Konflikt als potenzielle Ursache des Hemispasmus facialis konnte nicht identifiziert werden.

**Figure 5:**
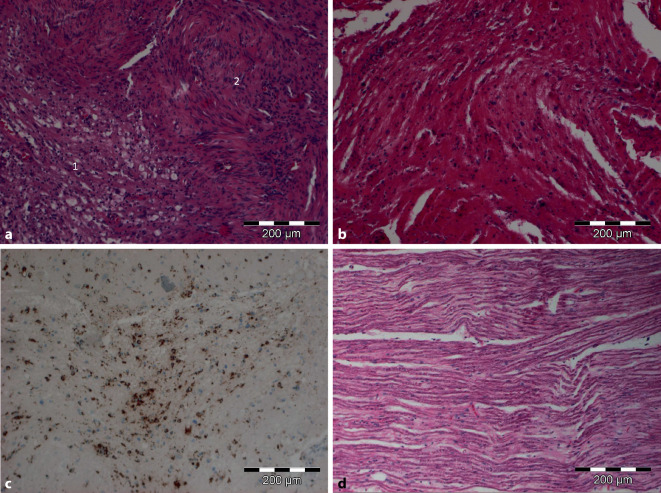

Histologisch wurde ein Vestibularisschwannom WHO I mit älteren Blutungen beschrieben. In der histologischen Aufarbeitung des N. vestibulocochlearis fanden sich Zeichen der Demyelinisierung, ödematöse und degenerative Veränderungen mit reduzierter Nervenfaserdichte und Makrophageninfiltraten (Abb. 5).

**Figure 6:**
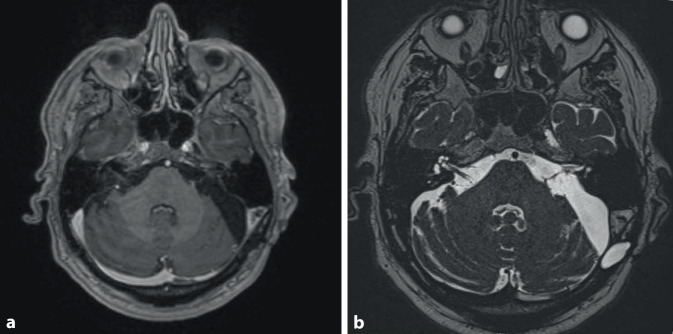

Postoperativ kam es zunächst zu einer ausgeprägten fazialen Parese (House & Brackmann Grad IV), welche sich im Verlauf sehr gut erholte (House & Brackmann Grad I). Die Hypästhesien V2 und V3 sowie der Hemispasmus facialis zeigten sich komplett regredient. Subjektiv habe sich ebenfalls sich Schwindelsymptomatik gebessert. MRT-Verlaufskontrollen über bislang 63 Monate bestätigen eine vollständige Tumorresektion (Abb. 6).

## Diskussion

Bei intrameatalen Vestibularisschwannomen stellen radiochirurgische Therapien wie Gamma-Knife oder CyberKnife etablierte Modalitäten dar, welche in den meisten Fällen eine Tumorkontrolle ermöglichen, gute Chancen eines Hörerhalts haben und nur selten mit Komplikationen verbunden sind [1, 3]. Eine operative Versorgung nach radiochirurgischer Vorbehandlung ist nur in wenigen Fällen nötig [9].

Dieser Fall zeigt, als bisher einziger, eine direkte Korrelation zwischen der postradiogenen Hörverschlechterung und dem histologischen Nachweis einer Demyelinisierung mit chronisch degenerativen Veränderungen des N. vestibulocochlearis. Bereits präoperativ war in der MRT-Bildgebung auffällig, dass sowohl der N. vestibulocochlearis als auch der N. facialis an Volumen zugenommen haben.

Nach radiochirurgischen Eingriffe sind Komplikationen wie Hypästhesie des N. trigeminus, Hemispasmus facialis oder Hörminderung bereits regelmäßig beschrieben [2, 5]. Dass diese Effekte auf eine Demyelinisierung durch die vorherige Therapie zurückzuführen sind, konnte bisher jedoch nicht gezeigt werden. Dass Demyelinisierung infolge einer Strahlentherapie auftreten kann und dieser Effekt dosisabhängig ist, konnte bei Tierversuchen gezeigt werden [11].

Bildgebend kam es bei dem gezeigten Fall nach der Gamma-Knife-Therapie zu einem Progress des kontrastmittelaufnehmenden Tumors im Kleinhirnbrückenwinkel. Differenzialdiagnostisch muss neben einem reinen Tumorprogress auch eine maligne Transformation in Betracht gezogen werden, welche in Einzelfällen nach vorheriger Bestrahlung beobachtet werden konnte [7]. Hinweise für Malignität ergaben sich bei unserem Fall nicht.

Neben dem Hörerhalt ist das Vorhandensein und die Ausprägung einer fazialen Parese ein wichtiger postoperativer Aspekt. Typischerweise ist hier das Outcome nach vorheriger Bestrahlung als schlecht einzustufen [4], allerdings konnten wir im vorliegenden Fall postoperativ eine sehr gute Erholung der Parese beobachten. Dies wird möglicherweise durch die perioperative Gabe von Nimodipin begünstigt. Es gibt Hinweise, dass Nimodipin neuroprotektive Eigenschaften besitzt und einen positiven Einfluss auf die Myelinisierung zu haben scheint [6, 8].

Dieser Fall zeigt erstmals ein morphologisches Korrelat für unerwünschte Effekte nach radiochirurgischer Therapie eines Vestibularisschwannoms und weist darauf hin, dass trotz einer gewissen räumlichen Distanz zum eigentlichen Tumor mit degenerativen Veränderungen nervaler Strukturen zu rechnen sein kann.

Generell empfehlen wir eine Behandlung von Vestibularisschwannomen im Fall eines nachgewiesenen Wachstums, es sei denn die Tumoren sind bei Diagnosestellung bereits als Koos 3 oder 4 klassifiziert. In solchen Fällen besteht eine Indikation zur Operation. Bei Tumoren bis Koos 2 ist alternativ eine strahlentherapeutische Behandlung möglich, bei Patienten unter 50 Jahren favorisieren wir auch in diesen Fällen eher den operativen Eingriff, wobei in jedem Fall ein Behandlungskonzept interdisziplinär (HNO, Neurochirurgie und Strahlentherapie) und individuell erstellt werden sollte.

## Fazit für die Praxis

- Radiochirurgische Therapien wie Gamma-Knife oder CyberKnife sind etablierte Modalitäten, welche in den meisten Fällen eine Tumorkontrolle und gute Chancen auf der Hörerhalt erlauben.
- Trotz sorgfältiger Therapieplanung müssen bei der Beratung der Patienten auch seltene Komplikationen berücksichtigt werden.
- Radiochirurgische Eingriffe können zu chronisch degenerativen Veränderungen und einer Demyelinisierung angrenzender neuraler Strukturen führen.
